# Addressing patient treatment preferences at trial recruitment

**DOI:** 10.1186/1745-6215-12-S1-A124

**Published:** 2011-12-13

**Authors:** Nicola Mills, Julia Wade, Athene J Lane, Freddie C Hamdy, David E Neal, Jenny L Donovan

**Affiliations:** 1 School of Social and Community Medicine, University of Bristol, Bristol, UK; 2 Nuffield Department of Surgical Sciences, University of Oxford, Oxford, UK; 3 Department of Oncology, University of Cambridge, Cambridge, UK

## Background

Patient recruitment is one of the main challenges in conducting randomised controlled trials (RCTs). Patients’ treatment preferences are viewed as a barrier to RCT recruitment yet there is little research to understand them. This study explored the expression of preferences by potential trial recruits, the response to them by recruiters, and their influence on trial participation decisions during trial recruitment appointments.

## Methods

We undertook an analysis of audio recordings of consecutive recruitment appointments to a UK multi-centre RCT of three different treatments for prostate cancer (the ProtecT - Prostate cancer testing and Treatment - study) over a three month period. 93/108 appointments with men aged 51-70 years were recorded successfully and analysed using techniques of content and thematic analysis.

## Results

Most potential participants expressed a desire for a particular treatment early in appointments, with their desires ranging on a continuum from hesitant to well-formed opinions. Recruiters explored these initial treatment views in the context of evidence-based treatment and study information which resulted in many men becoming uncertain about their initial views and open to RCT recruitment, often accepting a different treatment from their original ‘preference’. Only a quarter of men who initially expressed a wish for a particular treatment sustained or developed a clear treatment preference as the consultation proceeded and ultimately received this treatment. In most of these cases the recruiters established the rationale for the preference then provided specific information that counter-balanced their reasoning by emphasising the position of clinical equipoise, uncertainty of the prognosis and the pros and cons of their desired and non desired treatment. This counter-balancing of information continued until they were sufficiently satisfied that the man was making a fully informed decision.

## Conclusions

Many potential trial recruits will present initially with a treatment preference at trial recruitment but most of these preferences will dissolve after thorough exploration and targeted evidence-based information enabling trial participation. Only a minority of preferences are upheld after specific counter-balancing of information is tactfully given. The key for future research is to continue developing strategies that sensitively elicit and explore treatment preferences so that the more robust preferences can be distinguished from the ephemeral views to maximise trial recruitment.

